# Malnutrition, sarcopenia and cachexia: exploring prevalence, overlap, and perceptions in older adults with cancer

**DOI:** 10.1038/s41430-024-01433-9

**Published:** 2024-04-05

**Authors:** Alex F. Bullock, Michael J. Patterson, Lewis W. Paton, David C. Currow, Miriam J. Johnson

**Affiliations:** 1 Wolfson Palliative Care Research Centre, Hull York Medical School, University of Hull, Hull, UK; 2 Hull York Medical School, University of York, York, UK; 3 Faculty of Science, Medicine and Health, University of Wollongong, Wollongong, NSW Australia

**Keywords:** Geriatrics, Nutrition

## Abstract

**Background:**

Older adults with cancer are a growing population requiring tailored care to achieve optimum treatment outcomes. Their care is complicated by under-recognised and under-treated wasting disorders: malnutrition, sarcopenia, and cachexia. We aimed to investigate the prevalence, overlap, and patients’ views and experiences of malnutrition, sarcopenia, and cachexia, in older adults with cancer.

**Methods:**

Mixed-methods study with cross-sectional study and qualitative interviews. Interviews were thematically analysed through a phenomenological lens, with feedback loop analysis investigating relationships between themes and findings synthesised using modified critical interpretative synthesis.

**Findings:**

*n* = 30 were screened for malnutrition, sarcopenia, and cachexia, *n* = 8 completed semi-structured interviews. Eighteen (60.0%) were malnourished, 16 (53.3%) sarcopenic, and 17 (56.7%) cachexic. One or more condition was seen in 80%, and all three in 30%. In univariate analysis, Rockwood clinical frailty score (OR 2.94 [95% CI: 1.26–6.89, *p* = 0.013]) was associated with sarcopenia, reported percentage meal consumption (OR 2.28 [95% CI: 1.24–4.19, *p* = 0.008]), and visible wasting (OR 8.43 [95% CI: 1.9–37.3] *p* = 0.005) with malnutrition, and percentage monthly weight loss (OR 8.71 [95% CI: 1.87–40.60] *p* = 0.006) with cachexia. Screening tools identified established conditions rather than ‘risk’. Nutritional and functional problems were often overlooked, overshadowed, and misunderstood by both patients and (in patients’ perceptions) by clinicians; misattributed to ageing, cancer, or comorbidities. Patients viewed these conditions as both personal impossibilities, yet accepted inevitabilities.

**Conclusion:**

Perceptions, identification, and management of these conditions needs to improve, and their importance recognised by clinicians and patients so those truly ‘at risk’ are identified whilst conditions are more remediable to interventions.

## Introduction

Malnutrition, sarcopenia, and cachexia are three conditions that may be seen in older adults with cancer. All three conditions are associated with being unfit for, or having poorer tolerance to anti-cancer treatments, increased length of hospital stay, and poorer survival [1–4]. Malnutrition affects 20–85% of people with cancer, depending on diagnosis [5]. Similarly, sarcopenia and cachexia affect 15–50% and 25–80% of people with cancer, respectively [6]. However, we do not know how these conditions overlap in older adults with cancer, who are at risk of all three, or how best to identify the conditions in routine care and tailor management. There is no tool that simply attributes the relative contribution of each condition to a person with cancer’s overall health.

Despite their high estimated prevalence, and known impacts on patients’ health and quality of life [1–4], identification and subsequent treatment of these conditions is challenging. Although the impact of nutritional screening on clinicians’ time has been investigated (~6–12 min per patient) [7], we know little of the impact of nutritional screening, or screening for sarcopenia and cachexia, on patients, or of their knowledge of, views and experiences of the three conditions, of sarcopenia and cachexia in particular. Our previous work, looking at views and experiences of nutritional screening found that, although nutritional screening is seen as acceptable, patients’ misunderstanding and poor knowledge regarding aetiology and impact of malnutrition resulted in reduced risk perception and disbelief or disregard of nutritional screening results [8]. This review raised questions regarding perceptions and impact of malnutrition, and other nutrition-related condition upon high-risk populations, i.e., older adults with cancer, and the effectiveness of screening in clinical practice.

Overall body weight loss is a key clinical feature of both cachexia and malnutrition, with a loss of muscle mass also seen in all three conditions [1, 9, 10]. However, management of these three conditions varies; physical rehabilitation for sarcopenia may stabilise muscle mass, medical management of cachexia where the underlying cause can be treated, and dietary interventions for malnutrition [11–13]. To ensure the most appropriate treatment for each, the ability to distinguish conditions with their relative contributions, where more than one is present, is required.

Therefore, we conducted a mixed-methods study to explore the feasibility, and clinical utility of screening for malnutrition, sarcopenia and cachexia in a group of older adults with cancer, and explore patients’ views and experiences of the three conditions.

## Methods

This mixed-methods study had a convergent parallel design; cross-sectional data collection and qualitative interviews. The study was approved by the Central London Research Ethics Committee (reference 19/LO/1479), with the study performed in accordance with ethical standards laid down in the 1964 Declaration of Helsinki and its later amendments, with informed consent obtained from all participants.

### Participants

Participants were recruited between January 2020 and March 2020, from an inpatient tertiary cancer centre in the North East of England. Eligible patients were; older adults (aged ≥70 years) diagnosed with breast, colorectal, lung, prostate, head and neck, or upper gastrointestinal cancers, able to provide informed consent. Those considered by the multi-disciplinary team to be in the last few weeks of life, or those with insufficient English to provide fully informed consent or comply with study assessments, in the absence of suitable translation services, were excluded. Convenience sampling was used for participant recruitment.

### Data collection and analysis

#### Quantitative

Participants were screened by one researcher (AB) for malnutrition, sarcopenia and cachexia, using the SARC-F [14], European Working Group on Sarcopenia in Older People 2 algorithm (EWGSOP2) [10], the Mini Cachexia screening tool (MCASCO) [15]), and malnutrition screening tools Malnutrition Universal Screening Tool (MUST) [16], Patient Generated Subjected Global Assessment (PG-SGA) [17], and 3-Minute Nutrition Screening (3-MinNS) [18]. High inter-rater reliability has been seen with the MCASCO, SARC-F, Rockwood Clinical Frailty Scale, used to assess frailty, and functional measures including the timed up and go and sit to stand [15, 19–21]. Additional patient demographics, clinical characteristics and measures were recorded at baseline, including additional clinical assessments of frailty (Rockwood [22]), comorbidity (Charlson comorbidity index [23]), and recording of social and medical history. Equipment used included the portable Tanita BC545N BIA scale, Jamar Hydraulic Handgrip Dynamometer, elbow flexed to 90’c, forearm and wrist neutral. Tests included: sit to stand; crossed arms, 5 repetitions, timed up and go; seated to stand, walk 3-metres, turn and return to seated, chair-stand test; arms-crossed, 5 repetitions, mid-arm circumference; midpoint of acromion and olecranon process, with definitions for thresholds as per the EWGSOP2 guidelines [10].

Descriptive statistics, odds ratios, and univariate regression analysis were used. Univariate analysis was used to identify key predictor variables of the three conditions. Bonferroni correction was used to adjust for multiple testing. Analysis was performed using STATA SE17 [24]. Additional outcomes, of feasibility of assessments and study recruitment, and completion of physical markers, were also recorded.

#### Qualitative

Semi-structured face-to-face and telephone interviews were conducted with a subset of study participants. A topic guide (see Supplementary Material One) developed from the literature, patient public involvement, and team expertise, was used to explore patients’ experiences, views, and understanding of malnutrition, sarcopenia, cachexia, and the screening processes. Interviews were audio-recorded, transcribed verbatim, and analysed using thematic analysis [25] with a phenomenological lens, to focus on patients’ experiences [26]. NVivo 12 [27] software was used, with 50% of interviews double-coded (25% MP, 25% MJ). Following thematic analysis, data were subjected to loop analysis; of identifying relationships between themes, which, when merged, produced a loop diagram. Core steps of conducting loop analysis include; (1) identifying data sections for the arguments and their supporting rationales, (2) identifying relationships between variables or themes, (3) producing simple diagrams to represent each theme and their relationships, and (4) merging simple diagrams into a collective feedback loop diagram [28, 29], illustrated using Matchware Mindview 6.0 software [30].

A modified critical interpretive synthesis [28, 29] of overall study findings was conducted, by assembling ‘synthetic constructs’, of producing a reduced account of the context of all studies i.e., ‘summing up’, then creating a ‘synthesising argument’ in a framework that represents each construct and details the relationships between them [30].

## Results

### Quantitative results

See Table 1 for participant characteristics; *n* = 30 participants, median age 76.5 years (range 70–83 years), 72.2% male, *n* = 26 (76.9%) lived with a partner, were frail (mean Rockwood 4.1), with multiple conditions (mean Charlson comorbidity index score 8.1). Most common diagnoses were upper gastrointestinal (33.3%) and lung cancers (26.7%), with over half (59%) having localised disease.

**Table 1:** Participant demographics and clinical characteristics.

Age: (years) median (Standard Deviation), Range	76.5 (4.2), 70–83
Sex: Male/Female	21/30 (70% M)
Cancer diagnosis	
Breast	5
Lung	8
Prostate	3
Colorectal	3
Head and Neck	1
Upper gastrointestinal	10
Metastatic cancer	14 (46.7%)
Non-metastatic cancer	16 (53.3%)
**Anthropometrics** — Mean, SD, Range, % completion	
Weight, kg	76.5 (17.0), 40.9–112.9
	29/30 (96.7%)
Body mass index, kg/m^2^	25.4 (3.5), 16.0–34.6
	29/30 (96.7%)
Mid-arm circumference, cm	27.6 (4.3), 17.5–34.0
	28/30 (93.3%)
Hand grip strength, kg	21.8 (7.7), 7.0–39.0
	26/30 (86.7%)
Chair stand test, repeats	10.4 (4.8), 6–20
	10/30 (33.3%)
Timed up and go test, seconds	12.4 (3.2), 8.4–16.9
	9/30 (30%)
Appendicular skeletal muscle, kg	22.5 (4.6), 14.7–32.2
(from BIA)	12/30 (40%)
Skeletal muscle index, kg/m^2^	7.5 (1.3), 5.6–9.8
(from BIA)	12/30 (40%)
**Biochemical markers** — Mean, SD, Range of days from collection, % completion	
Albumin	2.3 (2.5), 0–10 days
	29/30 (96.7%)
Haemoglobin	2.2 (1.9), 0–7 days
	29/30 (96.7%)
C reactive protein	3.2 (3.2), 0–11 days
	26/30 (86.7%)
Lymphocyte count	2.2 (1.9), 0–7 days
	28/30 (93.3%)
**Clinical scales** — Mean, SD, Range, % completion	
Rockwood clinical frailty scale	3.9 (1.4), 1–9
(1—very fit, 9—terminally ill)	30/30 (100%)
Charlson comorbidity index	8.0 (2.4), 5–14
(mild 1–2; moderate, 3–4; and severe, ≥5)	30/30 (100%)

#### Feasibility of screening

Screening questions had minimal missing data (0.55%). Completion rates were high for all biochemical markers (66.7–100%) and most anthropometric measures (height, weight, body mass index (BMI) (96.7%), midarm circumference (93.3%), and hand-grip strength (86.7%)). Chair-stand test (33.3% complete), timed up and go test (30.8%), and bioelectrical impedance analysis (BIA) (41.7%) completion were low, with participants declining or unable to complete these measures.

#### Condition prevalence and overlap

Prevalence of malnutrition, sarcopenia and cachexia varied by screening tool or diagnostic criteria. For the malnutrition screening tools, prevalence varied between 39.3% (MUST) and 43.3% (3-MinNS) for severe risk of malnutrition, and 53.6% (MUST) to 76.7% (PG-SGA) for moderate to severe risk. For cachexia, prevalence was between 55.5% (MCASCO) and 56.7% (Fearon criteria), and sarcopenia, 48.2% (EWGSOP2) to 66.7% (SARC-F).

**Fig. 1:**
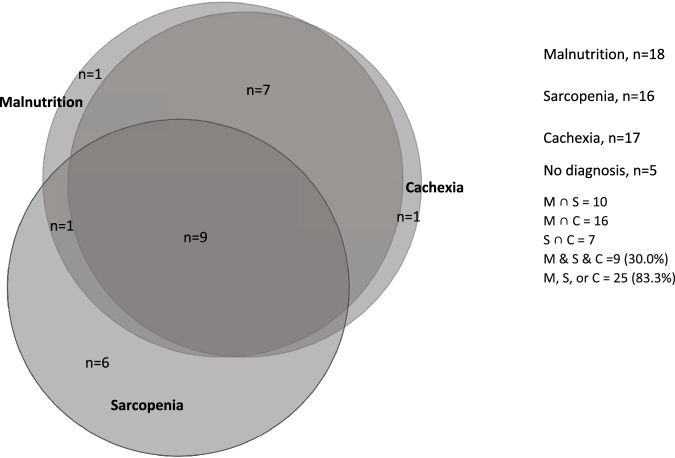
Venn diagram of the overlap of malnutrition, sarcopenia, and cachexia. The figure illustrates the overlap of each of the three conditions, with 83.3% of participants (*n* = 25) identified as having at least one of the three conditions. With this, 26.7% (*n* = 8) were identified as having only one of the conditions, 56.7% (*n* = 17) with two or more, and 30.0% (*n* = 9) were identified as having all three conditions. ∩ = intersection.

In total, 83.3% of participants were identified as having at least one of the three conditions, of which, 26.7% were identified as having only one condition, 30.0% were identified as having all three conditions (Fig. 1).

Of those who had localised disease (*n* = 16), *n* = 5 (31.3%) had evidence of severe malnutrition, *n* = 6 (37.5%) had evidence of sarcopenia, and *n* = 9 (50%) had evidence of cachexia.

A statistically significant overlap was seen between severe malnutrition and cachexia (OR: 28.8 [95% CI: 2.91–284.76], *p* = 0.004). When including moderate and severe risk of malnutrition, this relationship remained highly significant (OR: 88 [95% CI: 7.08–1094], *p* = <0.0001). No statistically significant relationships were seen between malnutrition and sarcopenia (OR 1.8 [95% CI: 0.41–7.81] *p* = 0.433), or between sarcopenia and cachexia (OR: 0.96 [95% CI: 0.23–4.10], *p* = 0.961).

Looking at baseline clinical characteristics, a strong positive relationship was seen between Timed Up and Go and Skeletal Muscle Index (correlation 0.8554, *p* = 0.0141), BMI and mid-arm circumference (correlation 0.785, *p* = <0.0001), and Charlson Comorbidity Index score and chair stand test (correlation 0.788, *p* = 0.0068). Moderate positive relationship was seen between Rockwood score and timed up and go (correlation 0.697, *p* = 0.0368).

#### Streamlining screening

Results of earlier systematic reviews [8, 31], alongside qualitative findings, were used to identify potential predictor variables for the univariate analysis. Table 2 displays odds ratios from univariate logistic regression analysis, of variables predicting each of the three conditions, with Bonferroni corrections also presented. BMI was a statistically significant predictor (OR: 0.78, [95% CI: 0.61–0.98], *p* = 0.04) of malnutrition, as were percentage meal consumption (OR: 2.28 [95% CI: 1.24–4.19], *p* = 0.008), appetite (OR: 2.21 [95% CI: 1.16–4.20], *p* = 0.015), and sunken temples (OR 8.43 [95% CI: 1.9–37.3], *p* = 0.005). Following a Bonferroni correction for multiple testing, only sunken temples remained significant.

**Table 2:** Univariate logistic regression of candidate predictors of key patient characteristics.

Predictor variable	Odds ratio	95% confidence intervals	*p* value	Multiple correction (Bonferroni)
**Severe malnutrition**				
Body mass index	0.78	0.61–0.98	*p* = 0.039	*p* = 0.31
Percentage meal consumption	2.28	1.24–4.19	*p* = 0.008	*p* = 0.06
Appetite	2.21	1.16–4.20	*p* = 0.015	*p* = 0.12
Sunken temples	8.43	1.9–37.3	*p* = 0.005	*p* = 0.04
**Sarcopenia**				
Hand-grip strength	0.75	0.60–0.94	*p* = 0.015	*p* = 0.12
Rockwood	2.94	1.26–6.89	*p* = 0.013	*p* = 0.10
**Cachexia**				
Appetite	1.85	1.01–3.39	*p* = 0.048	*p* = 0.38
Percentage monthly weight loss	8.71	1.87–40.60	*p* = 0.006	*p* = 0.05

Appetite (OR: 1.85 [95% CI: 1.01–3.39], *p* = 0.048) and percentage monthly weight loss (OR: 8.71 [95% CI: 1.87–40.60] *p* = 0.006), were significant predictors of cachexia, with percentage monthly weight loss remaining significant after multiple correction.

When predicting sarcopenia, both hand-grip strength (OR 0.75 [95% CI: 0.60–0.94], *p* = 0.015), with an approximate 25% decrease odds for every 1 kg increase in hand-grip strength, and Rockwood score (OR 2.94 [95% CI: 1.26–6.89] *p* = 0.013) were statistically significant predictors in univariate analysis, but did not remain significant following Bonferroni correction.

### Qualitative findings

Eight participants (75% male, median age 75 years), participated in interviews. Four major themes were generated; (1) *Dissonance*, a misalignment, or disagreement, in participants’ beliefs, and contradictions in their views and opinions regarding the role, and impact of the three conditions, (2) *Diagnostic overshadowing* was seen when symptoms relating to these conditions were attributed to the cancer or its treatment, or other issues, (3) *Between a rock and a hard place*, nutrition and physical function remained overlooked until weight loss impacted upon treatment options, however participants faced difficulties having concerns heard, and (4) *Study screening* was seen as a positive intervention and a gateway to help, but screening was often not conducted, or acted upon. Summarised in Table 3. Full details of the thematic analysis will be presented elsewhere.

**Table 3:** Main themes, sub-themes and data codes.

**Theme One**:	**Understanding of malnutrition, sarcopenia, and cachexia**		
**Dissonance**	Lay understanding of malnutrition, sarcopenia, and cachexia (macro)		No, not heard of that (sarcopenia) before (Pt1)
			Q: …it’s the word cachexia. A: No, nah you’ve missed me again (Pt5)
	Detachment of self-view		It’s not, it’s not something that I take seriously, because it’s not going to happen to me. Yeah (Pt8)
	Impact of malnutrition, sarcopenia, cachexia (macro) noticeable		When I could just get out of a chair, getting up, getting showered, getting dressed, wiped me out for the rest of the day (Pt1)
	Expected management		You need physiotherapy, you need work, you need to muscle to move again, cos if you don’t do that you stiffen up, and that makes it worse, so that’s what I want, I want Action (Pt8)
			Make sure you eat as nutritionally as you can, which will help you feel better, an cope with the treatment better. I think that’s a bit of common sense as well but erm I think they are helping you think you know it is important to keep, erm eating as healthy as you can (Pt3)
			Well, posh meal somewhere, or, or something like that, anything just to perk things up a bit (Pt8)
	Function as a priority		Getting showered, getting dressed, wiped me out for the rest of the day, and you know, it was just, or we planned to go somewhere and you know I would just, say I couldn’t come! (Pt1)
	Motivators to, and barriers for change		Somebody tried to, they came in and tried to but I think at that time I wasn’t very receptive anyway (Pt4)
	**Perceptions of risk**		
	Confidence in past health	Er, no cos I’m a very good eater, or I was (Pt8)	
		No, not really, as, as I think I’ve told you, I was, big, pretty fit, so… erm, didn’t go into it about… (Pt7)	
	Unhelpful generic advice	Yeah, they have said well try and do as much as you can, when you can (Pt3)	
		You know, make sure you can eat as healthily as you can, er (Pt3)	
	Opposing macro and micro views of nutrition and weight loss—Contradictory inevitabilities	Well, it’s (feeding tube) keeping me living, for starters (Pt5)	
		Well, I suppose it’s all part and parcel int it, really, I mean luckily I can eat, so I won’t ever get, er, malnutrition type of thing, but er, if it got worse, then you would (Pt5)	
**Theme Two:**	Overlooked and underplayed: cancer and treatment as priority	I call it a fitness test, and the results go to the consultant who’s supposed to do the surgery, and that will decide if I am fit enough to have the operation. So that is what I am aiming for (Pt2)	
**Diagnostic overshadowing**	Always comes back to the cancer	Sometimes it’s as though cancer is your first thought about everything, and I thought there’s life beyond that (…) you know coming along in the car I said you know that’s all we’ve talked about, we’re an hour away from here, about treatment and what the futures going to hold (Pt8)	
	Nutrition and function disregarded by clinicians	And we… were… not fobbed-off that’s too strong a word, but nothing really materialised… except we were able to see [DIETITIAN] and from that point on things, things have happened since (Pt1)	
	Explaining unexplained weight loss	Well that, well that’s inevitable really I think everybody eventually succumbs to that process (Pt8)	
		You’re compensating for your growing old aren’t you? (Pt8)	
	Not a medical problem	Yeah I think that they thought that they were concerned about us as a person, rather than us as a patient, our wellbeing was as important as the treatment they were giving (Pt3)	
**Theme Three**:	Weight loss noted	Erm and then I only noticed, you [HUSBAND] said that I was getting thinner, and then all of a sudden *I* noticed, and when you notice yourself that you are… (Pt1)	
**Between a rock and a hard place**	Rapid and visual weight loss	And it was, it was quite severe, so… we got, we went off to see the doc (Pt4)	
		I realise when, when things like, well the biggest thing that prompted me was when my wedding ring fell off, and, ha, I thought that’s a bit strange [laugh] (Pt7)	
	Difficulties raising and talking about weight loss	Erm, well erm, been dreadful really, I mean frightening [cough], very frightening (Pt6)	
		I think it was just a question of persuading somebody that, er I thought the weight loss was quite dramatic… (Pt1)	
		The only frustration was this weight loss, lack of energy started to arise, just getting somebody to take it onboard which has now happened (Pt1)	
	Inevitability of weight loss/poor function (acceptance)	Resigned? You can’t help but be resigned, I mean, I can’t do the things that I used to do now, I’m reconciled to it, what I do say though is that I’ll make the best of what I’ve got left (Pt8)	
		Well it’s alright, it just realise that I can’t eat as much as I could (Pt7)	
	Screening as an outlet	I think in a way, it was at the back of my mind, and that’s why that, that sitting down and standing up (functional assessments), brought it, if you like, to my mind (Pt2)	
**Theme Four:**	Benefits from screening assessment	But it’s good having the advice [pause] rather than thinking about it, thinking am I gonna make things worse you know leaping up and down or whatever (Pt5)	
**Study screening**		Helps if you’re talking about it… it’s better than it kind of left in the dark and only me knowing (Pt5)	
		I don’t sort of find it intrusive, I don’t find it you know, sort of difficult (Pt1)	
	Screening issues (barriers)	No, not really, as, as I think I’ve told you, I was, big, pretty fit, so.. erm, didn’t go into it about… (Pt7)	
		Yeah my strength isn’t as good as it used to be, but luckily for me I was I was very strong to start with… yea… my legs are the weakest, er ya luckily I was strong when all of this hit me so hopefully I can fight back some way (Pt7)	

Following thematic analysis, to gain a deeper understanding of the role of the three conditions in a patient’s health pathway and further understand their experiences and how any issues could be addressed, a loop analysis, to investigate relationships between themes, was undertaken and presented here.

#### Feedback loops

**Fig. 2:**
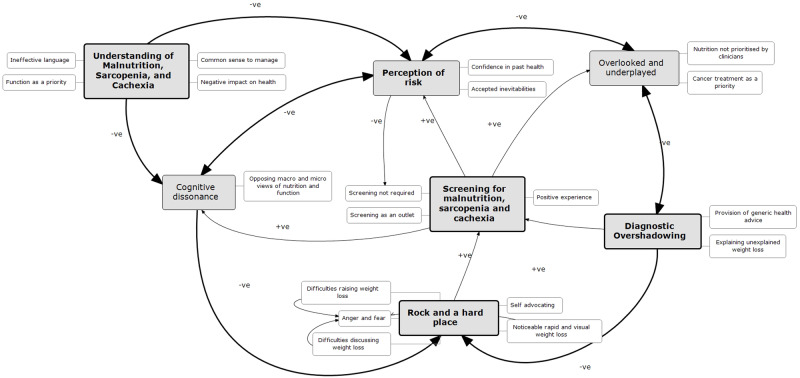
Feedback loop diagram illustrating interlinking themes of the views and experiences of malnutrition, sarcopenia, and cachexia in older adults with cancer. Loop 1: impact of misunderstanding, Loop 2: ending in a ‘rock and a hard place’, and Loop 3: the role of screening for malnutrition, sarcopenia, and cachexia. Loop 1 negatively impacts upon each of the associated themes (perceptions of risk, dissonance), which, alongside the impact of diagnostic overshadowing (loop 2)—both by patients and clinicians, terminates in patients being trapped between ‘a rock and a hard place’. However, screening for malnutrition, sarcopenia, and cachexia presented a possible solution to this (loop 3).

Three loops were generated from the qualitative thematic analysis. Figure 2 maps the relationships between themes, and the positive and negative feedback loops which influenced patients’ views and experiences.

#### Loop one: impact of misunderstanding

A lack of knowledge by participants regarding malnutrition, sarcopenia, and cachexia, and their causes and consequences, affected perceptions of risk of developing these problems. The assumed impact of the conditions on personal health was often minimised. This was despite acknowledgement that nutritional and functional problems may cause negative effects in others e.g., poorer health, reduced quality of life. Low perception of risk continued, despite nutritional and functional problems being viewed as a normal part of the cancer journey, and an expected part of ageing—exposing a dissonance in participants’ beliefs regarding nutrition and physical function, fuelled by a misunderstanding of the aetiology and potential severity of these conditions.

#### Loop two: ending in a ‘rock and a hard place’

Low perception of risk of developing these conditions was contributed to by diagnostic overshadowing [32]—with clinicians perceived as downplaying or disregarding concerns participants had regarding nutrition or physical function. Problems e.g., weight loss, were attributed the cancer, its treatment, other health problems, or ageing, by both patients, and in patients’ perceptions, by their clinicians, therefore were seen as expected, normalised and therefore disregarded. This disregarding of symptoms by clinicians confirmed to participants that these issues were minor, and therefore posed little risk to their health.

A belief that past positive health behaviours e.g., following a ‘healthy’ diet, or staying ‘active’, were protective against any future nutrition or functional problems, which was reinforced when participants received inadequate or unhelpful ‘generic’ nutrition or physical activity advice. Finally, the emotional, physical, and mental burdens that resulted from a cancer diagnosis were prioritised by participants over nutritional and/or physical function problems.

This overshadowing and misperception of risk, caused conflict when participants eventually became concerned with these problems. The ‘tipping point’ into concern appeared to be when visual changes e.g., rapid weight loss, or poor physical function were noticed, or endangered their chances of receiving anti-cancer treatments. This left participants at an impasse; realising their predicament with nowhere to turn to have their concerns addressed.

#### Loop three: the role of screening for malnutrition, sarcopenia and cachexia

Assessments for these conditions, completed as part of this study, were seen as acceptable. Screening presented an opportunity for participants to consider and raise concerns regarding their nutrition or physical function, in an environment where their concerns would not be disregarded or minimised. Screening could also be seen as an intervention in itself, with physical tests of function reassuring participants that they were able to complete basic movements. This suggested screening could act as an opportunity to positively affect each aspect of the loop diagram; with screening providing an opportunity to educate patients on these conditions, and provide an outlet for those worried but where their concerns had been overshadowed, as concerns regarding nutrition and physical function were actively sought and addressed. However, participant receptiveness to advice was affected by self-belief in current health; with confidence in past health behaviours and attributes preventing participants from believing screening is required.

## Discussion

This is the first study looking at the prevalence and overlap of malnutrition, sarcopenia, and cachexia in older adults with cancer, in addition, this is also the first study to investigate patients’ experiences of screening for sarcopenia and cachexia. Our study found a high prevalence, and substantial overlap, of all three conditions, with the overwhelming majority having evidence of one or more condition (83%) with a third diagnosed with all three. Despite their high prevalence and potential impact on tolerability of cancer treatment and daily function, these conditions were overlooked, underplayed, accepted as inevitabilities of ageing and disease, and yet also seen as personal impossibilities by patients.

Screening for malnutrition, sarcopenia and cachexia in hospitalised older adults with cancer was feasible and acceptable, however several measures of physical function were poorly completed with a significant proportion of patients declining or unable to comply. However, this in itself may be seen as a marker of risk; positive physical self-perceptions are known to be associated with increased physical activity, and conversely, fear of falling is a predictor of activity avoidance or restriction in older adults [33, 34], as well as the risk of future falls [35], and may correlate with actual performance, risk of sarcopenia, or low skeletal muscle mass [33, 34]. Further, visual markers, e.g., reduced portion sizes, reduction in physical capabilities, were recognised as important by patients. Our data supports streamlined screening for malnutrition, sarcopenia, and cachexia using reduced dietary intake and visible weight loss to identify malnutrition, Rockwood frailty assessment or handgrip strength for sarcopenia, and rapid weight loss for cachexia. However, due to the overlap of these conditions, and difficulty distinguishing cachexia from malnutrition in particular, differentiating between these conditions in a population at high risk of developing all three, to allow tailored care, remains a challenge.

Although malnutrition screening tools predominantly aim to identify ‘risk’ of malnutrition [16, 18, 36], their criteria for ‘risk’ often align with, or are identical to the diagnostic criteria for malnutrition [9, 37–39]. By contrast, screening tools for sarcopenia and cachexia aim to identify *established* conditions [10, 15]. Screening, surely, should identify patients at a stage when interventions may have more benefit, rather than identifying established conditions? Additionally, if these conditions continue to be viewed as accepted inevitabilities of cancer and ageing, confirmed by clinicians’ perceived disregard, then proposed management strategies may also be disregarded or under-prioritised.

### Strengths and limitations

Our mixed-methods approach, allowing triangulation of data and integration of findings enabled a richer understanding of this topic [40, 41], allowing increased confidence in our findings despite small sample sizes caused by difficulties in recruitment during the COVID-19 pandemic. The qualitative sample size target was based on information power [42]. As the topic explored was narrow and all participants had experiences of screening, data quality was rich despite the relatively small sample size, and allowed additional analysis using feedback loops. Concurrent data collection for interviews and screening with recent recall of the process allowed detailed exploration of participants’ views.

“The more recently published GLIM criteria [38] not included as a screening method for malnutrition within this study as GLIM criteria includes sarcopenia and cachexia within its diagnostic criteria. As discussed, the delineation and differentiation of malnutrition from sarcopenia and cachexia, to allow condition-specific treatment and management, is required. However, the overlap of the three conditions by the GLIM criteria hinders this, by conflating all nutrition-related wasting disorders, under ‘malnutrition’, which works against the need to distinguish between the three conditions [9, 43, 44].”

We recognise that there is overlap between these three wasting disorders and frailty, but in view of the identified gap in the literature regarding a tool to distinguish between them—compared with the well-validated assessments for frailty—our focus in this study was the wasting disorders. A robust exploration of the interplay with frailty was therefore outside our scope. However, further research is required to investigate the interplay of these conditions with frailty, given the findings of the importance of Rockwood for identifying sarcopenia.

Researching an understudied, and as seen, often overlooked topic, means this work has been able to provide a voice for this overlooked, but extensive group—of older adults with cancer and nutrition or physical function problems. Recruitment from a single site, focusing on six specific groups of cancer, makes the generalisability of these results limited. However, findings from more diverse study populations in prior systematic reviews [8, 45] support several findings of this mixed-methods study.

### Clinical and research implications

Greater acknowledgement and prioritisation of malnutrition, sarcopenia and cachexia by healthcare professionals is required when treating older adults with cancer. Disruption of the negative cycle of these problems and their symptoms being normalised or misattributed to other health conditions, and therefore accepted as normal and ignored, despite their negative impact upon patients, is required. This should be initiated by clinicians, including doctors, signalling the importance of this aspect of care.

Work is needed to adapt current screening tools to identify actual *risk* of developing these conditions, with a focus on signs, symptoms and clinical characteristics that predict risk, rather than established presence of the conditions. Additionally, differentiating between malnutrition and cachexia is not currently possible using current screening tools due to the overlapping diagnostic criteria, in particular the reliance on percentage weight loss to diagnose both, preventing tailored interventions.

The dissonance in patients’ views regarding their perceptions of the conditions—both perceiving themselves at no risk, whilst also seeing nutritional and functional problems as inevitabilities, requires further exploration to enable this to be addressed in clinical practice. With this, how to change clinicians’ perspectives of these conditions, and address ‘forgotten symptoms’, in this case, weight loss, reduced mobility, and related issues such as breathlessness and fatigue [46], requires work. This includes research into *how* to discuss nutritional and physical function problems with patients. The terms ‘malnutrition’, ‘sarcopenia’ and ‘cachexia’ are not understood by patients, and although ‘nutrition problems’ and ‘function’ are more readily comprehended, these did not always impart the potential seriousness of the conditions. Research into language and terminology used, and how to modify public health messages, is needed.

## Conclusion

Older adults with cancer have a high prevalence of malnutrition, sarcopenia and cachexia with overlap between them, particularly malnutrition and cachexia, resulting in burden, including poor tolerance of cancer treatment. Screening for all three conditions in this population is feasible and acceptable but only identifies established conditions rather than those at risk. Mobility-based physical measures are useful, and the ability to complete such measures may be considered an assessment in itself, with visual markers of change prioritised by patients.

We need to change perceptions and management of these conditions—raising their priority rather than allowing cancer, ageing, or multimorbidity to ‘overshadow’ these problems. Work is needed to determine how to identify and manage these conditions *before* they cause morbidity. Appropriate, well-conducted screening may provide a method to address these barriers, and provide patients with a positive experience and management of malnutrition, sarcopenia, and cachexia.

### Supplementary information

Supplementary Material One

## Data Availability

Further data may be available from the corresponding author upon reasonable request.
