# Supplementary material for: Malnutrition, sarcopenia and cachexia: exploring prevalence, overlap, and perceptions in older adults with cancer
Source: Eur J Clin Nutr. 2024 Apr 5;78(6):486–93. doi: 10.1038/s41430-024-01433-9 (PMC11182746; doi:10.1038/s41430-024-01433-9)
Supplement: Supplementary file 1 — Supplementary Material One [file 41430_2024_1433_MOESM1_ESM.pdf]

Online Supplementary Material One: Interview Topic Guide

Exploring patient views, experiences and understanding of assessments for malnutrition, sarcopenia and cachexia (MSC)

Aims and objectives

The central aim of this interview is to explore patient's views, experiences and understanding regarding assessments for malnutrition, sarcopenia and cachexia (MSC) following a cancer diagnosis

Main objectives to explore:

- The experiences of patient's regarding assessments for MSC
- The views of patient's concerning assessments for MSC
- Patient's understanding of assessments for MSC

Introduction
Aim: To introduce the research and set the context for the proceeding discussion.

- Introduce self and Hull York Medical School
- Introduce the study: who it is for and what it is about
- Talk through key points:
    - Purpose and length of interview
    - Any expenses refunded for travel
    - Voluntary nature of participation and right to withdraw
    - Recording of interview
- Confidentiality and how findings will be reported
- Any questions

Background and experience of cancer journey
Aim: To explore the experiences of cancer journey

"I have read your medical notes and know about your diagnosis and treatment so far, but I wonder if you could tell me about your experience in own words?"

- Elicit interactions with healthcare staff
    - Outpatient specialist, inpatient teams, clinical nurse specialists, allied healthcare professionals

Experiences of assessment for MSC
Aim: To explore the experiences of patient's regarding assessment for MSC

- For BOTH **nutrition** AND **activity levels or function**
    - "During your treatment / since your diagnosis, has anyone asked you about nutrition / activity or function"

    - If yes;
        - What were you asked?
        - Who asked you this?
        - How were you asked?
        - Why do you think you were asked?
    - If no; progress to next question

Views of assessment for MSC
Aim: To explore the views of patient's concerning assessment for MSC

- When asked about MSC:
    - How did you feel about being asked?
    - What was good?
    - What was bad?
    - What could be changed (if anything)?
        - Prompts: company, timing of assessment, who to conduct assessment, sense checking of questions

Patient's understanding regarding MSC
Aim: To explore patient's understanding of assessments for MSC

- "Has anyone mentioned to you…"
    - Malnutrition (problems with your eating)?
    - Sarcopenia (problems with muscle wasting or weakness)?
    - Cachexia (weight loss caused by cancer)?
- "What do you understand by these terms?"

"Do you think problems with MSC affect your health?"

- Physical
- Mental
- Social

Is there anything else you would like to mention?

Conclusion

- Thank participant for time
- Remind about confidentiality and anonymity
- Provide refund of travel expenses
